# *S*-Ketamine Mediates Its Acute and Sustained Antidepressant-Like Activity through a 5-HT_1B_ Receptor Dependent Mechanism in a Genetic Rat Model of Depression

**DOI:** 10.3389/fphar.2017.00978

**Published:** 2018-01-15

**Authors:** Kristian G. du Jardin, Nico Liebenberg, Manuel Cajina, Heidi K. Müller, Betina Elfving, Connie Sanchez, Gregers Wegener

**Affiliations:** ^1^Translational Neuropsychiatry Unit, Department of Clinical Medicine, Aarhus University, Aarhus, Denmark; ^2^Lundbeck US LLC, Paramus, NJ, United States; ^3^Centre of Excellence for Pharmaceutical Sciences, North-West University, Potchefstroom, South Africa

**Keywords:** serotonin (5-hydroxytryptamine, 5-HT), ketamine, antidepressants, forced swim test, CP94253, 5-HT_1B_ receptors

## Abstract

**Rationale:** The mechanisms responsible for the unique antidepressant properties of ketamine have only been partly resolved. Recent preclinical reports implicate the neurotransmitter serotonin [5-hydroxytryptamine (5-HT)] in the antidepressant-like response of ketamine, and modulation of 5-HT_1B_ receptors has been hypothesized to attain an important role.

**Objectives:** To evaluate the role of endogenous stimulation of 5-HT_1B_ heteroreceptors in the antidepressant-like activity of *S*-ketamine.

**Method:** Flinders sensitive line (FSL) rats, a genetic model of depression, were depleted of endogenous 5-HT by 4-chloro-DL-phenylalanine methyl ester HCl administration (pCPA; 86 mg/kg/day for 3 days). In pCPA-pretreated and control FSL rats, the acute and sustained effects of a single dose of *S*-ketamine (15 mg/kg) and the selective 5-HT_1B_ receptor agonist CP94253 (1–6 mg/kg) alone and in combination with *S*-ketamine were studied in the forced swim test (FST), a commonly used assay that detects antidepressant activity.

**Results:** pCPA pretreatment decreased cortical 5-HT levels to ∼6% but did not affect the baseline behavioral phenotype of FSL rats. *S*-ketamine demonstrated acute and sustained antidepressant-like activity, both of which were abolished by 5-HT depletion. Combining *S*-ketamine with a sub-effective dose of CP94253 (1 mg/kg) rescued *S*-ketamine’s acute and sustained antidepressant-like effects, when CP94253 was administered 2 h prior to the FST. Co-administration of *S*-ketamine and CP94253 did not affect the plasma level of either compound, suggesting that the observed behavioral interaction could not be ascribed to a kinetic drug-drug interaction.

**Conclusion:** 5-HT_1B_ receptor activation during testing appears to be critical for *S*-ketamine’s antidepressant-like potentials in this model.

## Introduction

Hours after a single i.v., infusion of the non-competitive *N*-methyl-D-aspartate (NMDA) receptor antagonist ketamine, patients with refractory major depressive disorder may experience a remarkable mood improvement (Berman et al., 2000). This rapid onset of antidepressant action is unlike the delayed effects of current-first line antidepressants, which require in the order of weeks to provide clinical efficacy. Moreover, the remission rate for ketamine may be up to 70% in patients that are resistant to current first-line treatments, and the mood improvement is typically maintained for more than a week after a single dose (Niciu et al., 2014). In animal models of depression, a single dose of ketamine induces an acute and sustained antidepressant-like response in line with clinical findings (reviewed in Browne and Lucki, 2013). Regrettably, ketamine may cause transient but serious psychotomimetic side effects and potentially also impact cognitive function. Ketamine is therefore not likely to gain widespread clinical use (Niciu et al., 2014). Nevertheless, the unique therapeutic properties of ketamine have prompted researchers to explore the mechanisms mediating its antidepressant effects in preclinical studies. Despite many efforts for more than a decade, no consensus on the mechanisms has been reached so far. In addition to modulation of glutamatergic neurotransmission, ketamine has been found to modify multiple aspects of serotonergic neurotransmission, including increased efflux in the prefrontal cortex (Amargós-Bosch et al., 2006; Yamamoto et al., 2013; Nishitani et al., 2014). Our group as well as others have shown that ketamine’s antidepressant-like activity in the forced swim test (FST) is abolished by serotonin [5-hydroxytryptamine (5-HT)] depletion induced by the irreversible tryptophan hydroxylase inhibitor 4-chloro-DL-phenylalanine methyl ester HCl (pCPA), which blocks the rate-limiting step of 5-HT synthesis (Gigliucci et al., 2013; Fukumoto et al., 2015; du Jardin et al., 2016). These data indicate that ketamine elicits its antidepressant-like actions via a 5-HT-dependent mechanism. Furthermore, a recent Positron emission tomography (PET) study in macaques demonstrated an association between ketamine treatment and 5-HT_1B_ receptor density in certain brain areas that may be important for its antidepressant activity (Yamanaka et al., 2014). The 5-HT_1A_ (Fukumoto et al., 2014), 5-HT_2_ (Fukumoto et al., 2014), and 5-HT_3_ (Kos et al., 2006) receptor subtypes have also been sparsely investigated in the context of ketamine’s antidepressant-like actions, but with limited indication that these receptor subtypes are implicated in the antidepressant-like effect.

We hypothesized that activity at 5-HT_1B_ receptors are essential for ketamine’s antidepressant-like activity in the FST. Studies were conducted in Flinders sensitive line (FSL) rats, a well-validated genetic rat model of depression, where depression-like behavior is observed in the FST compared to the control strain Flinders resistant line (FRL) rats (reviewed in Overstreet and Wegener, 2013). The acute and sustained antidepressant-like effects of *S*-ketamine in response to central 5-HT depletion and co-treatment with the selective 5-HT_1B_ receptor agonist CP94253 were evaluated in the FST. The degree of 5-HT depletion was determined in frontal cortical tissue homogenate using high performance liquid chromatography (HPLC), and 5-HT_1B_ receptor occupancy and drug plasma levels were determined by *ex vivo* autoradiography and mass spectrometry (MS), respectively.

## Materials and Methods

### Animals

Male Sprague-Dawley rats (10–12 weeks of age; 285–350 g; Taconic, Ejby, Denmark) and male Flinders line rats (FSL and FRL; 10–12 weeks of age; 280–350 g) from the colony maintained at Aarhus University (originally derived from the colony at the University of North Carolina, United States) were housed in pairs (Cage 1291H Eurostandard Type III H, 425 mm × 266 mm × 185 mm, Techniplast, Buguggiate, Italy) at 20 ± 2°C in a 12-h light/dark cycle (lights on at 07:00 am). The animals had *ad libitum* access to tap water and chow pellets. The animal colony was protected from outside noise, and all experimental procedures were performed in specially equipped rooms within the vivarium. The Danish National Committee for Ethics in Animal Experimentation had approved all animal procedures prior to initiation of the experiments (2012-15-2934-00254), and procedures were performed in compliance with Directive 2010/63/EU of the European Parliament and of the Council as well as with Danish Law and Order regulating animal experiments (LBK no. 253, 08/03/2013 and BEK no. 88, 30/01/2013).

### Drugs

pCPA was purchased from Sigma–Aldrich (St. Louis, MO, United States), the clinically used formulation of *S*-ketamine HCl (Pfizer, New York, NY, United States) was acquired via the Hospital Pharmacy of the Central Denmark Region, and H. Lundbeck A/S (Copenhagen, Denmark) kindly provided CP94253 HCl.

pCPA was dissolved in saline, whereas CP94253 and *S*-ketamine were dissolved in water. We administered all injections i.p., at a volume of 2.0 ml/kg. All doses are expressed as mg of base per kg body weight. Control animals were injected with vehicle at the time of a drug treatment.

### Behavioral Evaluation

#### Open Field Test

Locomotor activity was assessed in an open field in order to detect possible inhibitory or stimulatory drug effects that could confound the performance in the FST (procedure previously described in du Jardin et al., 2016). A squared open field arena (plastic; 50 cm × 50 cm × 37 cm) with a light intensity of approximately five lux was used. Each rat was allowed to move freely for 5 min. The test arena was thoroughly cleaned with 70% ethanol between each rat to minimize the impact of olfactory cues. A camera, located directly above the center of the field, recorded the session and the total distance moved was quantified using EthoVision XT video tracking software (version 11.0.928; Noldus Information Technology, Wageningen, The Netherlands).

#### Forced Swim Test

The antidepressant potential was assessed in a modified FST [Detke et al., 1995; specific procedure previously described in du Jardin et al. (2016)]. Since FSL rats inherently show a depression-like phenotype, no pre-swim session was required. The rat was placed in an acrylic plastic cylinder (60 cm in height, 24 cm in diameter) containing 40 cm of water (23°C) for 10 min. A camera, located directly in front of the cylinder, recorded the session. An experienced investigator blinded to the treatments measured the time spent struggling (defined as attempts to climb the cylinder wall or diving), swimming (defined as a forward propulsion in the water surface), and being immobile (defined as the absence of movements except for the necessary activities to keep the head above water).

#### Study Design

**FIGURE 1:**
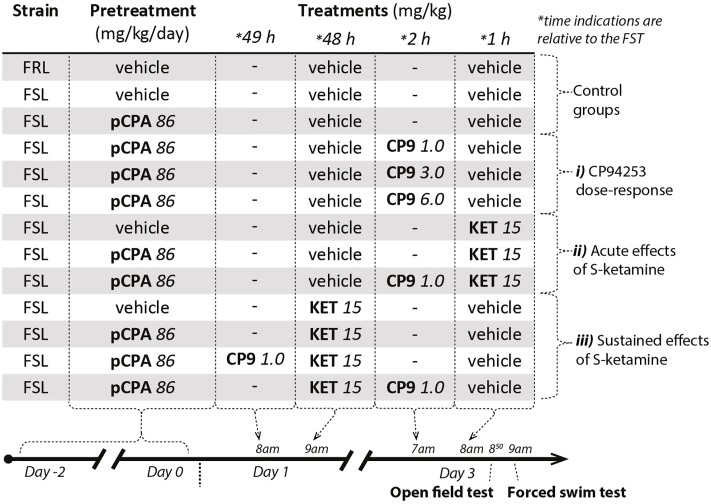
Experimental design and treatment groups. *CP9*, CP94253; *KET*, *S*-ketamine.

5-HT depletion was produced by pre-treating FSL rats with pCPA, 86 mg/kg/day, or vehicle for 3 consecutive days, as earlier shown to be an optimal depletion paradigm (Jensen et al., 2014). With the last pCPA injection as the reference time point, all FSL and FRL rats were subjected to the open field test at 71 h and 50 min and the FST at 72 h (**Figure 1**). Three experiments were undertaken (**Figure 1**):

(i) CP94253 dose-response experiment in the FST. 2 h prior to the FST, pCPA-pretreated FSL rats were injected with CP94253 at doses of 1, 3, or 6 mg/kg.

(ii) For pCPA- and vehicle-pretreated groups, the acute effects of *S*-ketamine were investigated by injecting rats with *S*-ketamine or vehicle 1 h prior to the FST. To assess the role of 5-HT_1B_ receptor agonism on *S*-ketamine’s acute antidepressant-like effect, *S*-ketamine (15 mg/kg) treatment in pCPA-pretreated FSL rats was preceded by a single dose of CP94253 (1 mg/kg) at 2 h prior to the FST.

(iii) For the sustained effects, *S*-ketamine (15 mg/kg) or vehicle was given 48 h prior to the FST to pCPA and vehicle-pretreated FSL rats. Furthermore, groups of pCPA-pretreated rats were co-treated with *S*-ketamine at 48 h before the FST as well as CP94253 (1 mg/kg) at 49 or 2 h prior to the FST.

To minimize the number of animals used, all rats received an injection with vehicle or *S*-ketamine at 2 or 48 h prior to the FST, which allowed us to use the same control groups for statistical analysis in all three experiments. However, vehicle injections were not given when rats were co-treated with CP94253 at 49 or 2 h prior the FST (addressed as a putative study limitation in the discussion). In total, the study comprised 11 different FSL treatment groups (**Figure 1**). In addition, a FRL control group injected solely with vehicle was included.

### Frontal Cortical 5-HT and 5-HIAA Levels

Twenty minutes after the conclusion of the FST, the rats were euthanized by decapitation for determination of frontal cortical levels of 5-HT and the 5-HT metabolite 5-hydroxyindole acetic acid (5-HIAA). The frontal cortices (20–40 mg wet weight) were dissected, flash frozen on powdered dry ice, and stored at -80°C. Subsequently, the right frontal cortices were mixed at a ratio of 1:5 (w/v) with 0.2 M perchloric acid and homogenized in 2 ml sample tubes for 2 s × 2 s with a probe sonicator (model UW2200; Bandelin Electronics, Berlin, Germany) at 70% power. The samples were then centrifuged at 4°C for 30 min at 21,000 ×*g*. The supernatant was transferred into Costar cellulose acetate filter tubes (0.22 μm; Corning, Inc., Corning, NY, United States) and centrifuged at 4°C for 10 min at 21,000 ×*g*. The solution was then transferred into HPLC sample vials for analysis of 5-HT and 5-HIAA levels. A Thermo Scientific HPLC system (Waltham, MA, United States) equipped with a Hypersil BDS C18 column, 3 μm particle, and 3 mm × 150 mm column provided separation of the analytes. Detection was carried out by a Thermo Scientific Dionex model 6011RS ultra Coulometric Analytical cell (E1: -150 mV; E2: +250 mV vs. a Pd reference electrode). Column and precolumn tubing were kept at 27°C while eluting the analytes with a MDTM mobile phase (Thermo Scientific Dionex Test Phase, 70-3829) at a flow rate of 0.5 ml/min.

### *Ex Vivo* Autoradiography: 5-HT_1B_ Receptor Occupancy

Behaviorally naïve male FSL rats were pretreated with pCPA, 86 mg/kg/day for 3 consecutive days. 70 h after the last pCPA injection, the rats were dosed with vehicle or CP94253 1, 3, or 6 mg/kg. At 72 h, the rats were euthanized by decapitation and the brains were collected, flash frozen on powdered dry ice, and stored at -80°C. At ∼1.2–1.5 mm anterior to Bregma, 20 mm thick coronal slices were cut at -20°C using a microtome cryostat. The sections were mounted on slides and stored with desiccant pellets at -20°C until use. Following a pre-incubation of 3 min (pre-incubation buffer: 170 mM Tris-HCl, 4 mM CaCl_2_, 0.01% L-ascorbic acid), the slides were incubated 1 h in assay buffer (170 mM Tris-HCl, 4 mM CaCl_2_, 0.1% L-ascorbic acid, 10 μM pargyline) including the 5-HT_1B_ receptor specific radioligand [^3^H]GR125743 (1 nM; Perkin Elmer, Boston, MA, United States). Non-specific binding was determined by adding 10 μM of the 5-HT_1B_ receptor specific ligand SB216641 to the assay buffer. After incubation with the radioligand, the slides were rinsed 2 min × 5 min in assay buffer (4°C) and briefly dipped in distilled water (4°C). Subsequently, the slides were air-dried for 30 min, transferred to a vacuum desiccator, and dried overnight. The slides were exposed to blank tritium storage phosphor screens (BAS-IP TR 2025 E, GE Healthcare, Buckinghamshire, United Kingdom) for 5 days at room temperature and subsequently processed in a BAS-5000 Phosphorimager (Fujifilm, Tokyo, Japan).

### *S*-Ketamine and CP94253 Plasma Levels

A cohort of male Sprague-Dawley rats was pretreated with pCPA, 86 mg/kg/day for 3 consecutive days. With the last pCPA injection as the reference time point, rats were single dosed with CP94253 or vehicle at 70 h as well as *S*-ketamine or vehicle at 71 h. All rats received at least one active treatment, resulting in three different treatment groups. At 72 h, the rats were euthanized by decapitation and blood was collected from the carotid arteries into EDTA tubes and kept on ice before centrifugation at 1,500 ×*g* for 10 min at 4°C. The supernatant was aliquoted and stored at -80°C.

Compound content in plasma samples were determined using an HPLC system (Aria TLX2; Thermo Fisher Scientific, San Jose, CA, United States) coupled with an MS system (TSQ Quantum Ultra; Thermo Fisher Scientific, San Jose, CA, United States). Prior to analysis, the samples were thawed at room temperature. An aliquot of 50 μl plasma sample was mixed with 150 μl dimethyl sulfoxide/acetonitrile (20:80) solution that contains an internal standard. The diluted plasma samples were then centrifuged (2,700 RPM; 15 min at 10°C), the supernatant removed and injected (10 μl) onto a liquid chromatography/MS/MS system for analysis. A Gemini column (Kinetex 2.6 μm C18, Å 50mm × 2.1 mm; Phenomenex, Torrance, CA, United States) was used for analytical separation. A typical 3-min gradient with the following mobile phases was used for separation: 0.1% formic acid in water (solvent A) and 0.01% formic acid in acetonitrile (solvent B). The mass spectrometer was equipped with a heated electrospray ionization probe, and the source conditions were as follows: Vaporizer temperature 450°C, spray voltage 3000, sheath gas at 40, ion sweep gas and auxiliary gas at 20, and capillary temperature at 300°C. Spectra was acquired in positive selected reaction monitoring mode with the parent masses of: 238.10 m/z and daughter ion 125.00 m/z at 35 collision energy and tube lens at 115 for *S*-ketamine as well as 258.16 m/z and daughter ion (1) 187 m/z at 20 collision energy, daughter ion (2) 229.00 m/z at 15 collision energy, and both tube lenses at 115 for CP94253.

### Data Analysis and Statistical Methods

Results are expressed as the mean ± standard error of the mean (SEM). Statistical significance was accepted at *p* < 0.05. GraphPad Prism version 6.07 (GraphPad Software, San Diego, CA, United States) was used for statistical analysis. All data sets were assessed for normality by manually inspecting Q–Q plots. Using Grubbs’ test, outliers were eliminated in cases where the normality assumption was reasonable with the exception of the potential outlier.

#### Behavioral Evaluation

Strain differences were analyzed using Student’s unpaired *t*-test, and drug effects were assessed by means of a two-way analysis of variance (ANOVA). In case of a significant treatment effect or interaction (pretreatment × treatment), appropriate Holm–Sidak’s multiple comparison tests were conducted. Because of the relatively low power of the analyses (*n* = 6–12 for experimental groups in the FST), we decided to only compare vehicle- and pCPA-pretreated control groups as well each experimental group with the relevant control group.

#### Frontal Cortical 5-HT and 5-HIAA Levels

Brain tissue data were normalized to the mean of vehicle-pretreated FSL control animals. The 5-HT turnover was defined as the ratio of the concentration of 5-HIAA and 5-HT (i.e., 5-HT turnover = [5-HIAA]/[5-HT]). The statistical analyses of 5-HT levels were identical with the procedure for behavioral evaluation. In the present experiment, frontal cortical 5-HIAA levels were too low for detection in pCPA-pretreated rats, and we therefore excluded these groups from the analyses of 5-HIAA and 5-HT turnover. Thus, any treatment effects on these parameters in vehicle-pretreated rats were investigated by use of a Student’s unpaired *t*-test.

#### *Ex Vivo* Autoradiography: 5-HT_1B_ Receptor Occupancy

**FIGURE 2:**
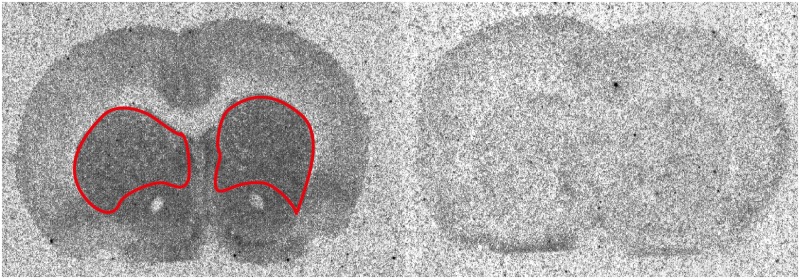
Coronal slices outlining the striatum at 1.5–1.2 mm anterior to Bregma were used for 5-HT_1B_ receptor occupancy assessments. These brain slices represent total (left) and non-specific binding (right). This region exhibited a high degree of specific binding.

Using the software ImageGauge 4.0 (Fujifilm) and a tritium micro scale standard slide (ART 0123-1EA, American Radiolabeled Chemicals, Inc., St. Louis, MO, United States), specific binding (total – non-specific binding) was determined for striatum (**Figure 2**) of three replicate slices. Specific binding levels for doses of CP94253 were expressed as a percentage of vehicle-treated rats. These percentages were subtracted from 100 to obtain percent receptor occupancy.

#### *S*-Ketamine and CP94253 Plasma Levels

Plasma levels of *S*-ketamine and CP94253 in Sprague-Dawley rats were analyzed using a Student’s unpaired *t*-test.

## Results

### Frontal Cortical 5-HT Levels and Turnover

No strain difference in frontal cortical 5-HT levels or turnover or 5-HIAA levels was found between vehicle-pretreated FSL and FRL control rats. Pretreatment with pCPA (86 mg/kg/day) for 3 consecutive days significantly decreased the 5-HT concentration to 6.0% in FSL control rats as demonstrated in the analysis of the CP94253 dose-response experiment [*F*(4,30) = 152.5; *p* < 0.001] as well as the acute [*F*(4,32) = 76.8, *p* < 0.001] and sustained [*F*(5,39) = 59.7, *p* < 0.001] effects of *S*-ketamine (data not shown). After pCPA pretreatment 5-HIAA levels were below the detection limit and therefore no data on 5-HIAA or 5-HT turnover are presented for these treatment groups. None of the drug treatments affected 5-HT levels, 5-HIAA levels, or 5-HT turnover relative to FSL control (data not shown; *n* = 7–8 for all groups).

### 5-HT_1B_ Occupancy after CP94253 Dosing

In FSL rats pretreated with pCPA, a single dose of 1, 3, and 6 mg/kg CP94253 at 2 h prior to euthanization resulted in a 5-HT_1B_ receptor occupancy of 45.1 ± 8.8, 75.0 ± 4.3, and 84.5 ± 0.5%, respectively, (*n* = 3 for all groups).

### *S*-Ketamine and CP94253 Plasma Levels

There was no statistical significant difference in CP94253 plasma levels between groups being co-administered vehicle (11.8 ± 4.0 ng/ml) and *S*-ketamine (6.3 ± 2.5 ng/ml). In *S*-ketamine-treated rats, co-treatment with CP94253 (69.1 ± 14.7 ng/ml) did not affect *S*-ketamine plasma levels compared to vehicle (89.8 ± 25.4 ng/ml; *n* = 6 for all groups).

### Open Field Test

Vehicle-pretreated FSL control rats (1851 ± 76 cm) displayed an average track length that was significantly higher than FRL controls (1613 ± 46 cm; *p* < 0.01). There were no drug-related effects on locomotor activity in any of the three drug studies, i.e., CP94253 dose-response [*F*(4,41) = 1.5; *n.s.*], acute *S*-ketamine ± CP94253 [*F*(4,44) = 0.3; *n.s.*], and sustained *S*-ketamine ± CP94253 [*F*(5,57) = 1.921; *n.s.*; Supplementary Figure S1].

### Forced Swim Test

**FIGURE 3:**
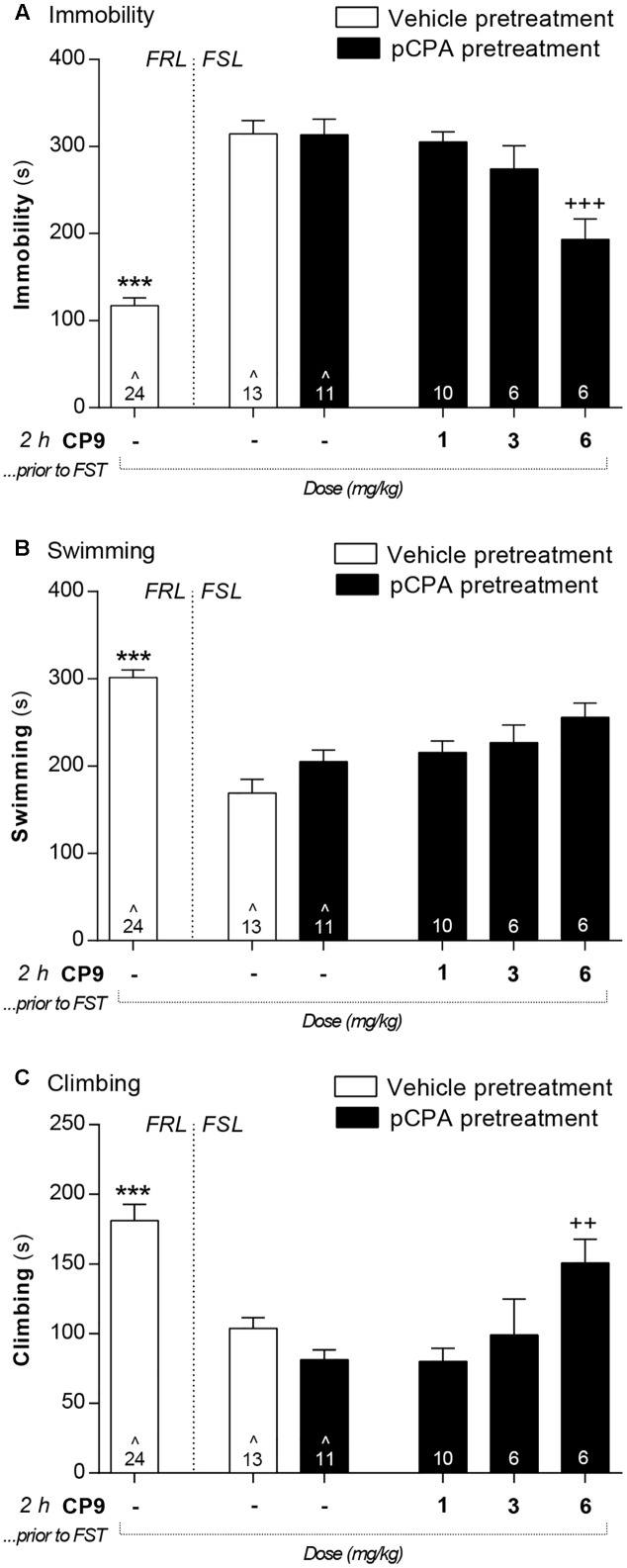
CP94253 displayed antidepressant-like activity in the FST. Vehicle-pretreated FSL control rats displayed a significantly higher immobility **(A)** as well as lower swimming **(B)** and climbing **(C)** behavior compared to FRL controls. pCPA pretreatment did not affect the behavior of FSL rats. When administered 2 h prior to the FST, CP94253 reduced immobility and enhanced climbing in 5-HT depleted FSL rats with a minimum effective dose of 6 mg/kg. Asterisks represent significant differences from vehicle-pretreated FSL control rats (^∗∗∗^*p* < 0.001). Plus signs represent significant differences from pCPA-pretreated FSL control rats (^++^*p* < 0.01; ^+++^*p* < 0.001). Values are mean ± SEM. The number of animals (*n*) is shown in each column. ˆ Data previously published in du Jardin et al. (2016).

**FIGURE 4:**
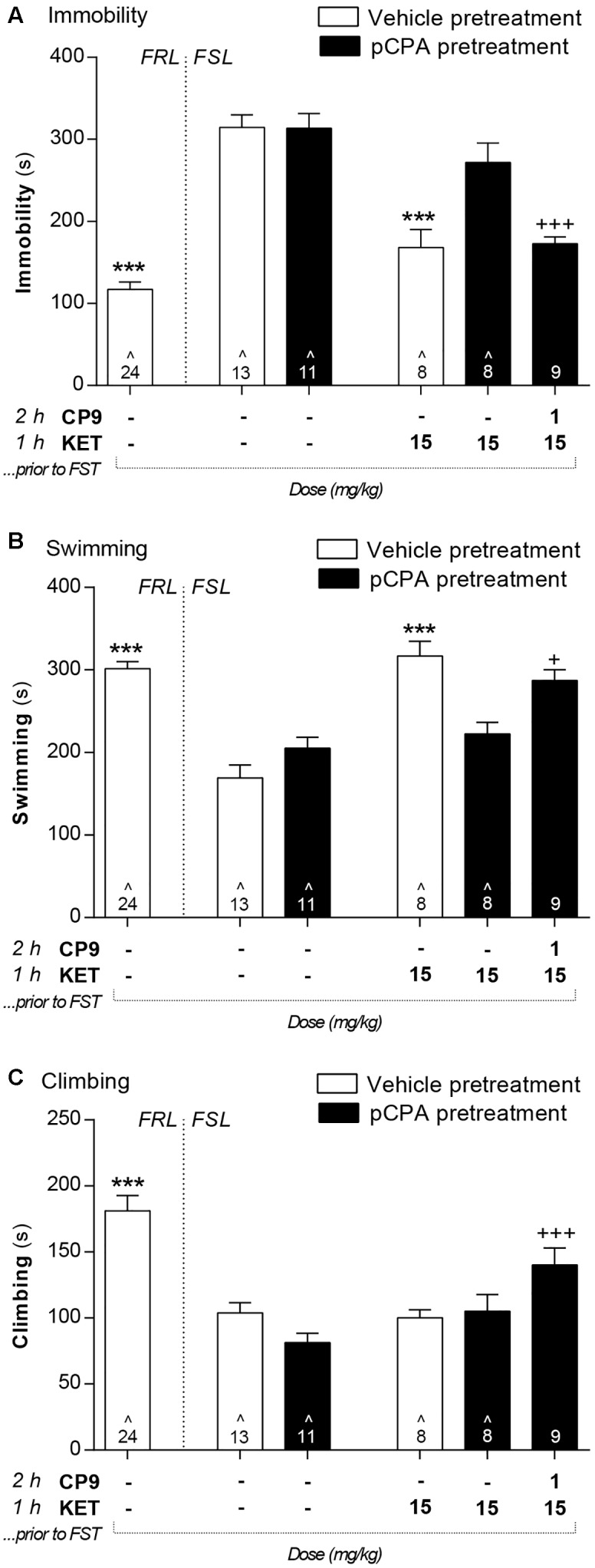
The acute antidepressant-like effects of *S*-ketamine were abolished by 5-HT depletion but rescued by the 5-HT_1B_ receptor agonist CP94253. In the FST, vehicle-pretreated FSL control rats displayed a significantly higher immobility **(A)** as well as lower swimming **(B)** and climbing **(C)** behavior compared to FRL controls. These behaviors were not affected by pCPA pretreatment. *S*-ketamine administration at 2 h before the FST decreased immobility and increased swimming in vehicle- but not pCPA-pretreated FSL rats. Supplementing this regimen with a 5-HT_1B_ receptor agonist 2 h prior to testing reduced immobility as well as increased swimming and climbing behavior relative to 5-HT depleted FSL control rats. Asterisks represent significant differences from vehicle-pretreated FSL control rats (^∗∗∗^*p* < 0.001). Plus signs represent significant differences from pCPA-pretreated FSL control rats (^+^*p* < 0.05; ^+++^*p* < 0.001). Values are mean ± SEM. The number of animals (*n*) is shown in each column. ˆ Data previously published in du Jardin et al. (2016).

**FIGURE 5:**
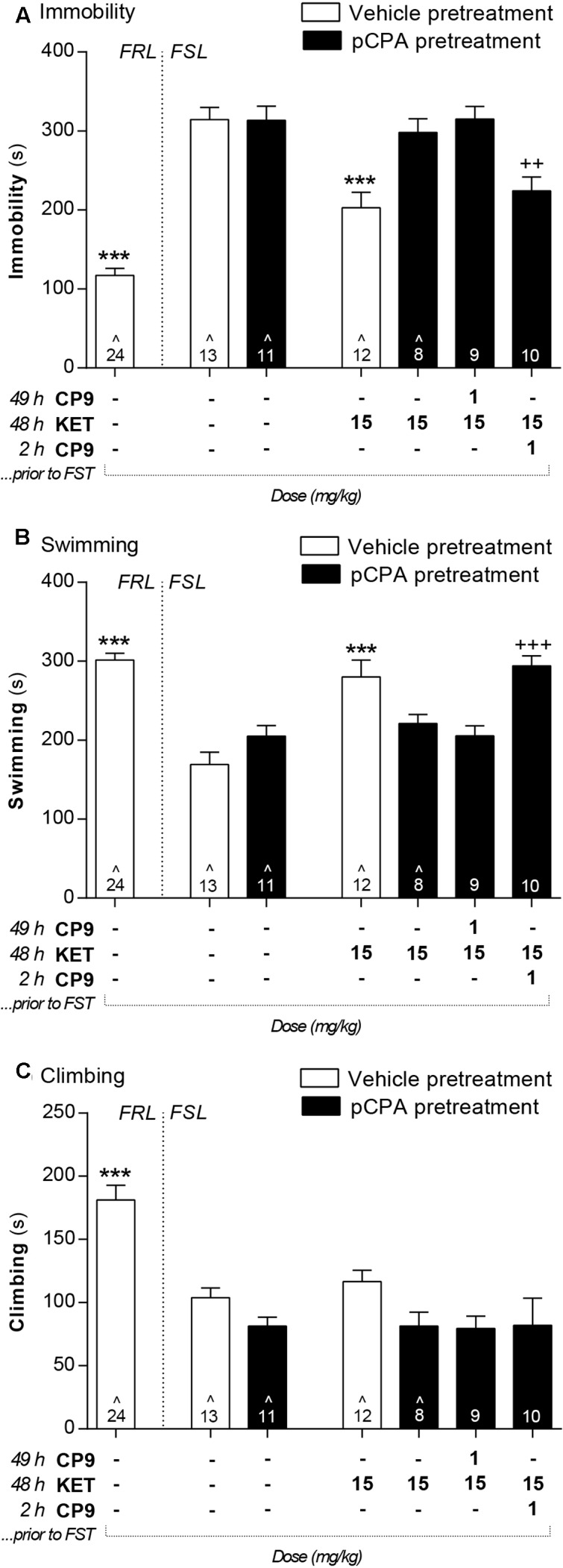
The sustained antidepressant-like effects of *S*-ketamine were abolished by 5-HT depletion but rescued by the 5-HT_1B_ receptor agonist, CP94253. In the FST, vehicle-pretreated FSL control rats displayed significantly higher immobility **(A)** as well as lower swimming **(B)** and climbing **(C)** behavior compared to FRL controls. 5-HT depletion did not affect the behavior of FSL control rats. In vehicle-pretreated FSL rats, *S*-ketamine administration at 48 h prior to the FST reduced immobility and increased swimming. These effects were abolished by pCPA pretreatment and restored by co-treatment with a 5-HT_1B_ receptor agonist at 2 h, but not 49 h, prior to testing. Asterisks represent significant differences from vehicle-pretreated FSL control rats (^∗∗∗^*p* < 0.001). Plus signs represent significant differences from pCPA-pretreated FSL control rats (^++^*p* < 0.01; ^+++^*p* < 0.001). Values are mean ± SEM. The number of animals (*n*) is shown in each column. ˆ Data previously published in du Jardin et al. (2016).

Vehicle-pretreated FSL control animals displayed a significantly higher immobility in the FST, whereas swimming and climbing were decreased compared to FRL control rats (**Figures 3**–**5**). In FSL control rats, no difference was found in any FST readout between pCPA and vehicle pretreatment for any analyses (*F*- and *p*-values for individual analyses are presented below).

In pCPA-pretreated FSL rats, a 6 mg/kg dose of CP94253 at 2 h prior to testing significantly reduced immobility [*F*(4,41) = 6.2; *p* < 0.001] and enhanced climbing behavior [**Figure 3**; *F*(4,40) = 4.7; *p* < 0.01]. Doses of 1 and 3 mg/kg did not affect these behaviors in 5-HT depleted FSL rats. There was an overall difference between treatment groups on swimming behavior [*F*(4,41) = 3.9; *p* < 0.01], but *post hoc* analyses did not identify any statistically significant differences.

Administration of *S*-ketamine (15 mg/kg) at 1 h prior to the FST significantly decreased immobility [*F*(4,44) = 16.9; *p* < 0.001] and increased swimming behavior [*F*(4,44) = 16.3; *p* < 0.001] compared to vehicle-pretreated FSL control rats (**Figure 4**). The *S*-ketamine-induced effects on immobility and swimming were abolished by pCPA pretreatment but rescued by co-treatment with CP94253 (1 mg/kg) at 2 h prior to the FST. Climbing behavior was not affected by *S*-ketamine treatment, but the combination of CP94253 at 2 h and *S*-ketamine 1 h prior to the FST enhanced climbing [*F*(4,42) = 5.0; *p* < 0.01] in pCPA-pretreated FSL rats.

At 48 h after *S*-ketamine treatment (15 mg/kg), vehicle-pretreated FSL rats displayed significantly reduced immobility [*F*(5,57) = 8.8; *p* < 0.001] and enhanced swimming behavior [*F*(5,57) = 9.9; *p* < 0.001] compared to FSL control rats (**Figure 5**). These effects were prevented by 5-HT depletion with pCPA, but again the addition of CP94253 at 2 h prior to testing rescued the effects. No effect was observed when *S*-ketamine and CP94253 were administered in combination at 48 and 49 h prior to the FST, respectively. Finally, there was no effect on climbing behavior in the analysis of *S*-ketamine’s sustained effects [*F*(5,56) = 1.9; *n.s.*].

## Discussion

In the current study, we assessed the role of 5-HT_1B_ receptor stimulation for the antidepressant-like action of *S*-ketamine.

In agreement with published data, vehicle-pretreated FSL control rats displayed increased immobility as well as decreased swimming and climbing compared to FRL controls in the FST. Importantly, these depression-like behaviors cannot be ascribed to decreased locomotor activity since FSL rats in fact displayed a longer track length in the open field test compared to FRL rats. Thus, we confirm that FSL rats constitute a valid preclinical model to study antidepressant-like potentials.

Pretreatment with pCPA for 3 consecutive days reduced the frontal cortical 5-HT levels to ∼6% of the vehicle control level. This reduction is comparable to previous reports using the same formulation in similar dosing regimens (Cryan et al., 2000; Prinssen et al., 2002; Lapiz-Bluhm et al., 2009; Jensen et al., 2014; Wallace et al., 2014). Although not supported by statistical analyses, it is reasonable to assume that pCPA pretreatment caused a profound reduction in 5-HIAA levels since it was not possible to detect this 5-HT metabolite in the frontal cortices of pCPA-pretreated animals.

Since the objective of the present study was to gain novel mechanistic insights into the antidepressant action of *S*-ketamine by evaluating its effects on behavior in response to manipulations of serotonergic neurotransmission, it was important that these manipulations were behaviorally neutral on their own. The profound 5-HT depletion did not modify the FSL rats’ behavior in the FST. These results are consistent with data from other groups, who do not demonstrate any changes to baseline behavior in the FST after pCPA pretreatment in rodents (Page et al., 1999; Harkin et al., 2003; Savegnago et al., 2007; Szewczyk et al., 2009; Gigliucci et al., 2013). Similarly, there was no effect of pCPA pretreatment on locomotor activity. Thus, any effects of pCPA on a treatment response in the FST were not confounded by changes in locomotor activity. Unlike the present findings, a previous report showed decreased track length following 5-HT depletion with pCPA (Jensen et al., 2014). However, in this study, the investigators used another strain and sex of rats as well as administration of pCPA for 4 consecutive days as opposed to three in the present study. It may therefore be speculated that methodological dissimilarities account for the differences between the two studies.

To exclude any effect of CP94253 alone, its minimum effective dose (MED) was determined in a dose-response experiment in pCPA-pretreated FSL rats 2 h before the FST. CP94253 did not significantly affect behavior at 1 or 3 mg/kg, but at 6 mg/kg reduced immobility and increased climbing behavior. This effect was not accompanied by any changes in locomotor activity. Thus, CP94253 displayed antidepressant-like activity in pCPA-pretreated FSL rats with a MED of 6 mg/kg. According to our *ex vivo* autoradiography data, this dose corresponds to a 5-HT_1B_ receptor occupancy of 84.5%. These data are consistent with previous reports, wherein the authors found that CP94253 with a MED of 5 mg/kg acutely reduces immobility in mice in the FST (Tatarczyñska et al., 2004, 2005).

Both 1 and 48 h after a single injection, *S*-ketamine (15 mg/kg) significantly reduced immobility and increased swimming behavior in vehicle-pretreated FSL rats. In line with previous reports (Garcia et al., 2008; Li et al., 2010; Liebenberg et al., 2015), these data suggest that a single dose of *S*-ketamine induces an antidepressant-like response within 1 h that is sustained for at least 48 h. 5-HT depletion induced by pCPA pretreatment prevented the acute and sustained effects of *S*-ketamine on immobility and swimming behavior. These data suggest that both the acute and sustained antidepressant-like effects of *S*-ketamine are mediated via a 5-HT-dependent mechanism.

The potential role of 5-HT_1B_ receptor stimulation in *S*-ketamine’s antidepressant properties was explored by combining *S*-ketamine treatment with a single dose of the selective 5-HT_1B_ receptor agonist CP94253 in 5-HT-depleted FSL rats. Although the immobility time after 3 mg/kg CP94253 was not significantly different from vehicle-treated rats, we decided to use 1 mg/kg dose as an add-on to *S*-ketamine treatment in order to minimize the risk of introducing a confounder. This dose corresponds to 45.1% 5-HT_1B_ receptor occupancy and can therefore be considered pharmacologically active. The acute antidepressant-like effect of *S*-ketamine was rescued in pCPA-pretreated rats by preceding treatment with CP94253 (1 mg/kg; 2 h prior to testing) as demonstrated by a significantly reduced immobility and enhanced swimming and climbing behavior in these animals. Likewise, CP94253 (1 mg/kg; 2 h prior to testing) significantly decreased immobility and increased swimming behavior in 5-HT-depleted FSL rats treated with *S*-ketamine 48 h prior to testing. In summary, these data suggest that administration 2 h prior to testing with a 5-HT_1B_ receptor agonist at a dose that was inactive by itself restored *S*-ketamine’s acute and sustained antidepressant-like potentials. Furthermore, we did not observe any effect of CP94253 when administered 1 h prior to a single dose of *S*-ketamine at 48 h prior to the FST. Although values were associated with some variation, co-administration of *S*-ketamine and CP94253 did not seem to affect the plasma level of either compound, suggesting that the observed behavioral interaction was not due to a kinetic drug-drug interaction. Thus, it appears that a certain tone at 5-HT_1B_ receptors during testing, but not during *S*-ketamine administration, is essential for the sustained antidepressant-like effect of *S*-ketamine.

5-HT_1B_ receptors function as inhibitory G-protein coupled autoreceptors and heteroreceptors (Sari, 2004). 5-HT_1B_ autoreceptors are located presynaptically on serotonergic neurons where they exert negative feedback by inhibiting 5-HT neuronal firing and release (Morikawa et al., 2000; Sari, 2004). However, during conditions where 5-HT tone is already very low, such as when 5-HT is depleted with pCPA, it is reasonable to assume that these receptors have negligible effects, and any effects produced by a 5-HT_1B_ receptor agonist are therefore most likely to be mediated by 5-HT_1B_ heteroreceptors. This notion is supported by the finding that a 5-HT_1B_ receptor agonist produces a stronger antidepressant-like effect in animals subjected to pretreatment with pCPA or 5,7-dihydroxytryptamine creatinine, which destroys 5-HT containing nerve terminals and thereby 5-HT autoreceptors (Chenu et al., 2008). Thus, the restoring effect of CP94253 on *S*-ketamine’s responses in the FST is plausibly mediated via 5-HT_1B_ heteroreceptor stimulation.

Post-synaptic 5-HT_1B_ heteroreceptors are co-localized with NMDA or α-amino-3-hydroxy-5-methyl-4-isoxazolepropionic acid (AMPA) receptors on dendrites (Peddie et al., 2008, 2010), and are therefore well positioned to modulate the putative targets of *S*-ketamine. Moreover, 5-HT_1B_ heteroreceptors have been demonstrated presynaptically in glutamate terminals of the dorsal and ventral striatum (Mathur et al., 2011), rendering 5-HT_1B_ receptor mediated modification of glutamate firing possible directly in the terminal. However, there is currently no clear mechanistic explanation for the involvement of 5-HT_1B_ receptors in the antidepressant action of *S*-ketamine. The nature of this mechanism may fit into either of two principal possibilities (or a combination of both; du Jardin et al., 2016): (i) activation of 5-HT_1B_ heteroreceptors may assume an essential, regulatory role for extra- or intracellular pathways that lead to *S*-ketamine’s antidepressant response; (ii) *S*-ketamine mediates its antidepressant effect via modulation of 5-HT_1B_ heteroreceptor signaling. In line with the latter possibility, it was found that ketamine increased 5-HT_1B_ binding in the nucleus accumbens, ventral globus pallidus, and nucleus reuniens of macaques in a recent PET study (Yamanaka et al., 2014). These upregulations were suppressed by the AMPA receptor antagonist 2,3-dihydroxy-6-nitro-7-sulfamoylbenzo(f)quinoxaline (NBQX), which is known to block the antidepressant-like effects of ketamine in the FST (Maeng et al., 2008; Autry et al., 2011), thereby implicating this finding in the mechanisms responsible for the antidepressant activity. Ketamine has been found to modulate activity in numerous molecular pathways, and studies investigating a potential interaction between these pathways and 5-HT_1B_ heteroreceptors may enhance our knowledge of the 5-HT_1B_ receptor dependent mechanism in the antidepressant effects of ketamine. For instance, increased brain-derived neurotrophic factor secretion (Autry et al., 2011) as well as enhanced eukaryotic elongation factor 2 (Autry et al., 2011) and mechanistic target of rapamycin (mTOR) (Li et al., 2010) phosphorylation have been reported following ketamine treatment, and a thorough evaluation of the dependency of these effects on activity at 5-HT_1B_ heteroreceptors may be one approach to gain novel insights. Additionally, studies exploring the effects of ketamine on 5-HT_1B_ receptor activity may also provide important information. Finally, the relationship presented here between 5-HT_1B_ heteroreceptor agonism and ketamine’s antidepressant-like activity should be validated in alternative model systems.

The current study has several limitations that should be noted. First, no control vehicle-injections were administered at the time of CP94253 dosing. Therefore, it is possible that the stress associated with an additional injection might have affected the behavior of rats treated with C994253. However, we find it unlikely that an additional injection should have a positive impact on the behavior in the FST and, thus, the antidepressant-like effects associated with CP94253 are plausibly mediated by this drug’s pharmacodynamics rather than any stress associated with an additional injection. Second, the affinity of *S*-ketamine at the NMDA receptor is twice as high than that of the racemic mixture frequently used in the literature (Ebert et al., 1997; Moaddel et al., 2013), and the isoforms may also exhibit different affinity for non-NMDA receptor targets (Hustveit et al., 1995). Thus, the *S*-ketamine dose used in the current study plausibly has a different pharmacological profile compared to that of a similar dose of racemate ketamine. Additionally, preclinical studies have reported differentiated antidepressant-like properties of *S-* and *R*-ketamine (Zhang et al., 2013; Yang et al., 2015), and hence, the data presented here may not apply to the R-enantiomer and the racemic mixture. Therefore, whether endogenous 5-HT receptor activation in this model is essential for the antidepressant-like activity of R-ketamine and the racemate needs to be empirically determined in future studies. Third, we used Sprague-Dawley rats to assess plasma levels of CP94253 and *S*-ketamine as opposed to FSL in the behavioral experiments. However, the FSL strain was bred from Sprague-Dawley rats, and therefore, we find it implausible that any kinetic drug-drug interaction is evident in one strain and not the other. Fourth, it must be acknowledged that depression is considered a multi-circuitry disorder (Crowell et al., 2014; Michèle and Giannis, 2015), and not only related to the function of cortical areas as studied in the present work. Future studies should establish the contributions and relevance of those networks to the questions addressed in the present work. Similarly, since the exact contribution to the neuronal circuitry of the 5-HT_1B_ receptor as either autoreceptor or heteroreceptor cannot be determined in the present work, future studies with 5-HT_1B_ transgenic mice, optogenetic techniques or DREADD should be employed.

## Conclusion

Profound 5-HT depletion with the irreversible tryptophan hydroxylase inhibitor pCPA did not alter the depression-like behavior of FSL rats in the FST. In the FST, *S*-ketamine had acute and sustained antidepressant-like effects, both of which were abolished by 5-HT depletion. In this context, the 5-HT_1B_ receptor may be of special interest since treatment 2 h prior to testing with the selective 5-HT_1B_ receptor agonist CP94253 at a dose that had no effect as a single treatment restored both the acute and sustained antidepressant-like effect of *S*-ketamine in 5-HT depleted rats. Importantly, the plasma levels of *S*-ketamine and CP94253 did not seem to be affected by co-administration of either compound, suggesting that the observed behavioral interaction was not mediated by changes in pharmacokinetics. Thus, a certain tone at 5-HT_1B_ heteroreceptors (pre-synaptically or post-synaptically) during testing appears to be critical for *S*-ketamine’s antidepressant-like potential.

## Author Contributions

KdJ, NL, MC, HM, BE, CS, and GW participated in the design of all experiments. KdJ, NL, and MC performed the experiments, assembled, analyzed and interpreted the data. All authors contributed to the writing of the article and have approved the final manuscript.

## Conflict of Interest Statement

KdJ has received travel grants from H. Lundbeck A/S. MC and CS were full time employees of Lundbeck US LLC at the time when the studies were undertaken. GW reported having received lecture/consultancy fees from H. Lundbeck A/S, Servier SA, AstraZeneca AB, Eli Lilly A/S, Sun Pharma Pty Ltd., Pfizer, Inc., Shire A/S, HB Pharma A/S, Arla Foods Amba., Alkermes, Inc., and Mundipharma International, Ltd., and research funding from the Danish Medical Research Council, Aarhus University Research Foundation [AU-IDEAS initiative (eMOOD)], the Novo Nordisk Foundation, the Lundbeck Foundation, and EU Horizon 2020 (ExEDE). The other authors declare that the research was conducted in the absence of any commercial or financial relationships that could be construed as a potential conflict of interest.
