# Supplementary material for: S-Ketamine Mediates Its Acute and Sustained Antidepressant-Like Activity through a 5-HT1B Receptor Dependent Mechanism in a Genetic Rat Model of Depression
Source: Front Pharmacol. 2018 Jan 15;8:978. doi: 10.3389/fphar.2017.00978 (PMC5775507; doi:10.3389/fphar.2017.00978)
Supplement: Supplementary file 1 [file Data_Sheet_1.docx]

**Supplementary Material**


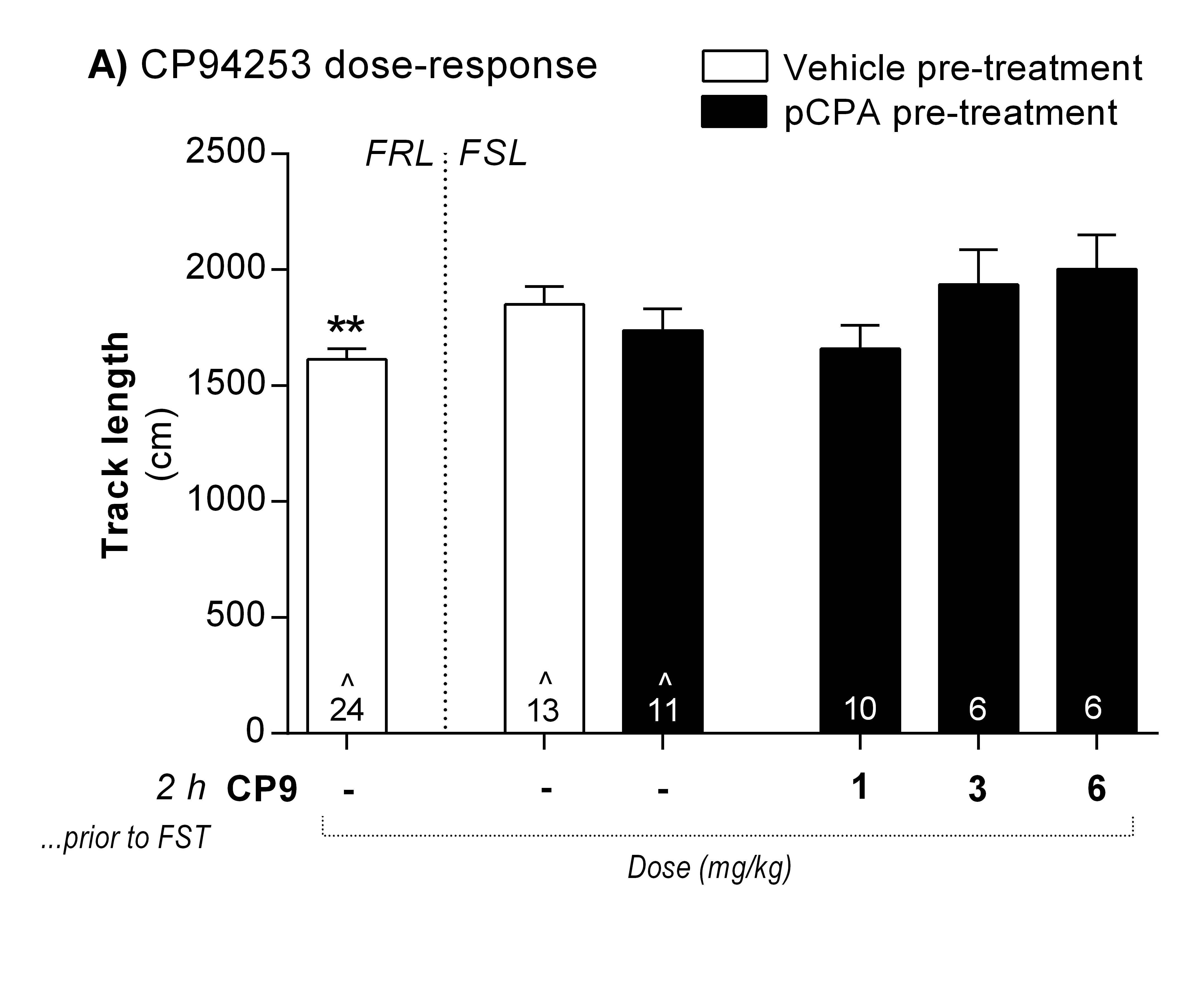

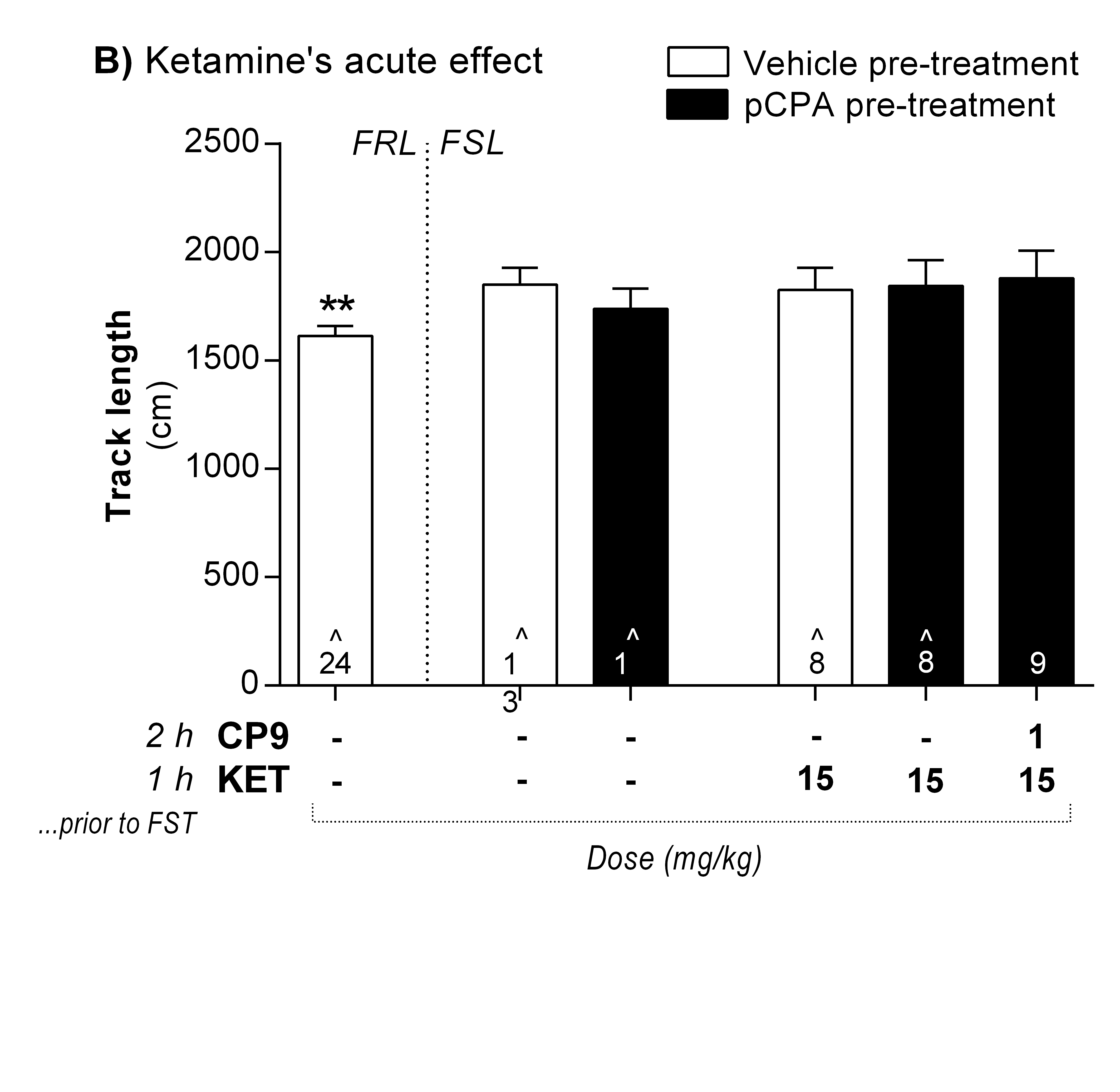

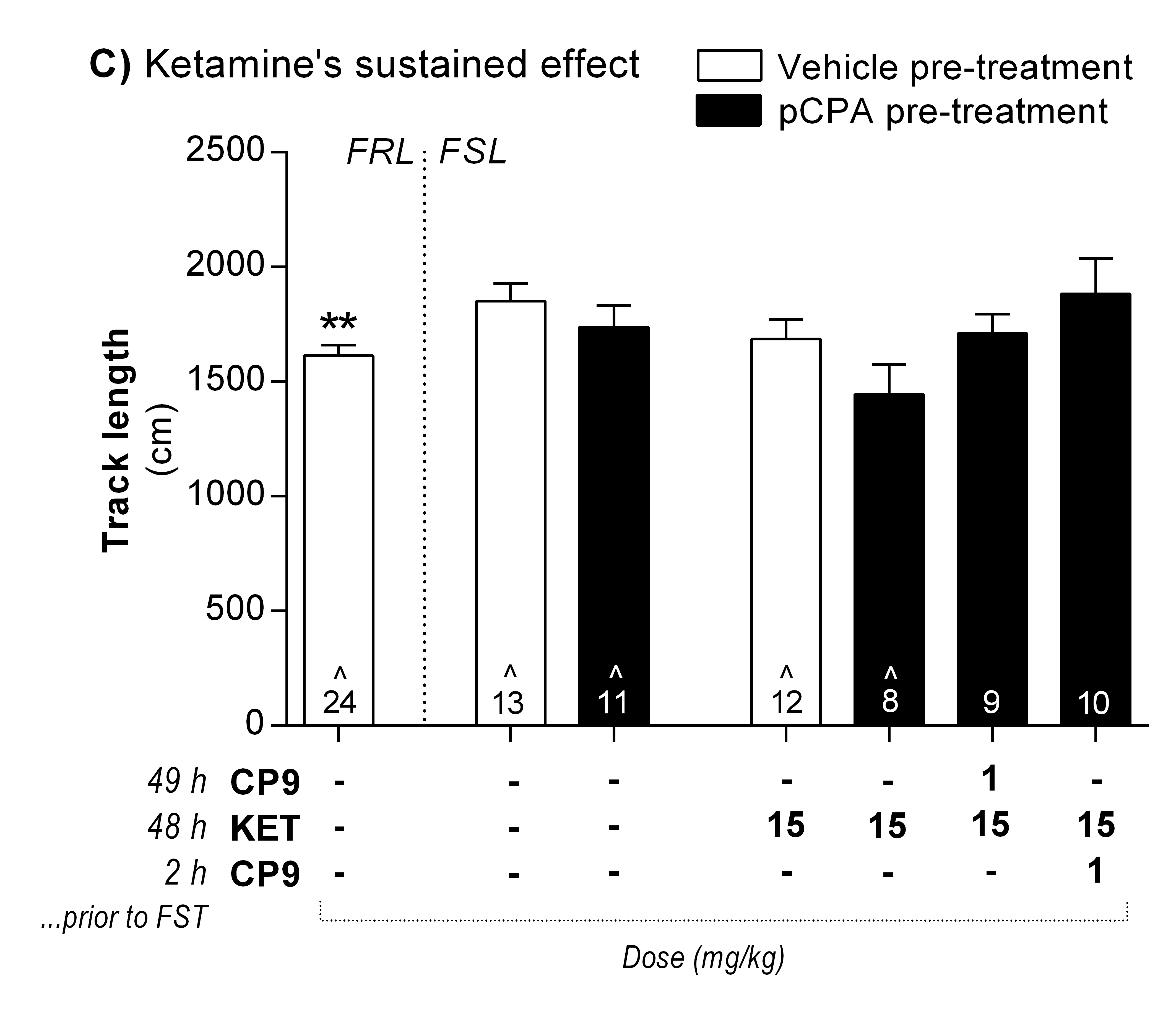


**Fig. S1** Locomotor activity was unaffected by drug administrations. Compared to FRL control rats, vehicle pretreated FSL control rats exhibited a significantly higher track length in the open field test, which was not affected by pretreatment. There were no drug-related effects on locomotor activity in any of the three drug studies, i.e., CP94253 dose-response (panel A), acute S-ketamine ± CP94253 (panel B), and sustained S-ketamine ± CP94253 (panel C). Asterisks represent significant differences from vehicle pretreated FSL control rats (***p*<0.01). Values are mean ± SEM. The number of animals (*n*) is shown in each column. ^data previously published in (du Jardin et al. 2016)
